# Swedish Clinicians' Knowledge, Practices, and Access to Structured Guidance Regarding Maladaptive Exercise in Eating Disorders

**DOI:** 10.1111/sjop.70102

**Published:** 2026-04-14

**Authors:** Elin Monell, Emma Forsén Mantilla

**Affiliations:** ^1^ Department of Clinical Neuroscience Karolinska Institutet Stockholm Sweden; ^2^ Department of Medical Epidemiology and Biostatistics Karolinska Institutet Stockholm Sweden; ^3^ Department of Physical Activity and Health The Swedish School of Sport and Health Sciences Stockholm Sweden

**Keywords:** clinicians' knowledge, eating disorders, guidelines, maladaptive exercise, work practices

## Abstract

Maladaptive exercise is a common and serious symptom in eating disorders (EDs), yet often poorly addressed in treatment. This study examined clinicians' knowledge, practices, and access to structured guidance such as clinic‐level practices/informal protocols related to maladaptive exercise, and whether these varied by profession, experience, or personal exercise habits. A cross‐sectional survey was completed by 102 clinicians working in Swedish ED care. The survey assessed self‐reported knowledge, clinical practices, and access to clinic‐level practices/informal protocols. Differences by profession, years of ED work experience, and personal physical activity were analyzed using Fisher's exact test and Spearman correlations. Most clinicians viewed maladaptive exercise as an aggravating symptom but reported limited formal training and moderate confidence in managing it. Assessment was common, but structured interventions were rare and opinions about them varied by profession. Only 42% reported access to clinic‐level practices/informal protocols, while 84% expressed a need for national guidance. Despite recognizing maladaptive exercise as clinically significant, ED clinicians report limited training and inconsistent guidance. Profession‐specific differences and the strong demand for national guidelines underscore the need for structured, evidence‐based approaches to both the assessment and treatment of maladaptive exercise, as well as adequate training for clinicians in their application.

## Key Points

- Maladaptive exercise is a clinically significant feature in eating disorders, and yet there are considerable differences in how it is defined, assessed, and managed within research and clinical practice.
- Swedish clinicians expressed uncertainty regarding how to define, assess, and manage maladaptive exercise and highlighted the need for clearer guidance and more standardized approaches.
- This study highlights that efforts to better define maladaptive exercise, as well as evidence‐based approaches for assessment and treatment of maladaptive exercise in eating disorders, are both relevant and requested by clinicians.

## 1 Introduction

Eating disorders (EDs) are serious psychiatric disorders associated with significant mental and physical impairments, long illness periods and high relapse rates, as well as great suffering for affected, their families and the society as a whole (Treasure et al. 2020; Van Hoeken and Hoek 2020). EDs are characterized by an anxious preoccupation with food, shape, and weight, and notably disturbed eating patterns including restriction and binge‐eating. Maladaptive exercise is a common and clinically significant symptom in EDs that has received far less attention than other behavioral symptoms (e.g., binge‐eating, self‐induced vomiting). Maladaptive exercise includes not only structured physical activity but also less formal, compulsive movement patterns. It is present in at least half of patients with ED, in all ED diagnoses, in males and females, and across the age span (Levallius et al. 2017; Monell et al. 2018). Maladaptive exercise is associated with higher risk for relapse, longer illness duration, longer periods of inpatient treatment, and less optimal treatment outcomes (Carter et al. 2004; Levallius et al. 2017; Liao et al. 2024; Monell et al. 2018; Smith et al., 2013). Even so, exercise has for long been neglected in both ED research and treatment methods. Further, definitions of maladaptive exercise in EDs differ, causing additional confusion, for instance in relation to screening and assessment (Schaumberg et al. 2026). Existing definitions include driven exercise, compensatory exercise, and compulsive exercise, all somewhat differing in their focus of specific characteristics of the activity (e.g., type, frequency, duration) and/or its associated psychological components (e.g., anxiety/guilt when not exercising, exercising to avoid negative emotions).

The diagnostic system DSM‐5 only mention exercise as an example of recurring inappropriate compensatory behaviors necessary for a diagnosis of bulimia nervosa (American Psychiatric Association 2013). No guidance of the characteristics of such exercise is offered, and no other kind of exercise is mentioned, which means that a wide range of clinically relevant behaviors and cognitions relating to exercise and physical activity may go undetected. While some well‐established national guidelines for the treatment of EDs (e.g., National Institute for Health and Care Excellence 2017) do acknowledge exercise‐related behaviors, they typically do not provide detailed, symptom‐specific guidance on how to assess or manage problematic exercise in clinical practice. Although there are examples of proposed definitions and guidelines for assessment and treatment of maladaptive exercise (e.g., Cook et al. 2016; Dittmer et al. 2018), none has yet been accepted and recommended in national treatment guidelines for wider implementation (see recent reviews: Hallward et al. 2022; Mathisen et al. 2023). In the most common standardized treatment methods for EDs (i.e., transdiagnostic CBT‐E for adults, and family‐based treatment for children; Fairburn 2008; Lock and Le Grange 2013), several interventions depend on individual symptomatology. These symptoms (binge‐eating, self‐induced vomiting, restrictive eating etc.) and accompanying interventions are described in detail in the manuals but mainly excluding maladaptive exercise (except for advising patients to stop excessive/compensatory exercise). Exercise restriction (i.e., refrain from exercise and avoid moving about excessively) remains the most common somewhat standardized intervention, particularly in medically unstable patients with anorexia nervosa. Further, although physiotherapists have often delivered body‐oriented treatments at ED units (e.g., body awareness, low intensity exercise, psychoeducation on healthy habits), inclusion, focus and content of such interventions are not fully standardized. Likewise, individual therapists may also include interventions targeting maladaptive exercise inspired by other treatment methods, but as such, they are lacking in standardization. Structured methods for exercise inclusion exists in the field (e.g., PED‐t; Mathisen et al. 2020), but they are not yet included as a standard treatment option. Thus, while some patients may receive at least a few interventions for their exercise behaviors, these interventions are likely dependent on factors such as clinic‐level practices, informal protocols, or locally developed routines (e.g., “no physical activity for patients with a BMI< 15” or “patients are allowed to take walks after x weeks in treatment”), the availability of physiotherapists, and clinician features (e.g., knowledge, formal education, confidence). There might also be an effect of clinicians' personal exercise habits (Davies et al. 2007). Notably, a recent study highlights that brief education can improve clinicians' self‐efficacy and perceived competence regarding maladaptive exercise, although their factional knowledge only improved moderately (Quesnel et al. 2025). The study also revealed difficulties in translating knowledge into clinical decision‐making which emphasizes the continued need for structured, interdisciplinary education and clear, structured, and symptom‐specific guidance.

Attitudes toward and management of maladaptive exercise were examined in 2009 at the top managerial level of 49 treatment units in Scandinavia and Great Britain (Bratland‐Sanda et al. 2009). Maladaptive exercise was generally regarded as a compulsive behavior, a dysfunctional affect regulation strategy, and a core ED symptom. The majority reported assessing maladaptive exercise at admission, two thirds reported including physical activity in treatment and/or psychoeducation, and all units reported having “guidelines” (likely clinic‐level routines/protocols) for how to manage maladaptive exercise, although no published versions of these (or other) guidelines were available. The countries were generally alike, although Sweden stood out in mainly considering BMI when applying exercise restriction. As such, ED treatment units in Scandinavia seem to have long been aware of maladaptive exercise as a symptom that should be assessed and managed, but with no consensus or common guideline or protocol as to how. Also, recent qualitative research highlights that Swedish patients would like a greater focus on problematic exercise in treatment, particularly interventions aiming for more healthy practices (Simon et al., 2024). Current treatment approaches were experienced as insufficient in how exercise was addressed, leaving patients feeling frustrated.

### 1.1 Aims

The overall aim of this study was to examine how maladaptive exercise is regarded by a wide range of ED clinicians. Specifically, we examined their subjective knowledge, clinical practices, and their perception of access to structured symptom‐specific guidance (e.g., clinic‐level practices/informal protocols) regarding maladaptive exercise. Lastly, we examined if these issues were influenced by profession, experience, or personal exercise habits.

## 2 Materials & Methods

### 2.1 Participants

Participants were 102 clinicians (82 women, 20 men; mean age = 45, SD = 10.7, range 26–65) working within Swedish ED care settings, which was the study inclusion criteria. A majority (49 participants) worked in specialized ED outpatient care, 13 in specialized ED day patient care, five in specialized ED inpatient care, and 20 worked in multiple specialized ED care settings (i.e., outpatient, day patient and/or inpatient care). Eleven worked in private practices, whereof six in combination with work in specialized ED care settings, and the remaining four worked with EDs in general psychiatry settings. 61% worked with adults (> 18 years), 23% with children/adolescents, and 16% with all age groups. Sixteen (of 21) of Swedish administrative geographic regions were represented; participants from the capital area dominated (25%). Most common professions within ED treatment settings were represented (see results).

### 2.2 Survey

The survey was developed by the authors, aided by a reference group comprised of researchers and clinicians, during the spring of 2023 (Data S1). It comprised 34 items aiming to capture participants': (1) knowledge of; (2) clinical practices for; and (3) access to clinic‐level practices/informal protocols for exercise/physical activity in EDs. For most items, answers are rated 1–5 (1 = not true 3 = partly to 5 = true). Items were created based on the authors' knowledge of the literature and experience from contact with clinicians regarding exercise in EDs. The survey also included demographic information, including profession, working experience within ED care, and personal exercise habits (structured exercise, everyday physical activity). The survey was anonymous and distributed using Google forms. All items were in Swedish (subsequently translated into English by the authors).

### 2.3 Procedure

The survey was distributed to members of the Swedish Eating Disorder Society (SEDS) at a member meeting in May 2023 with 244 registered participants (actual turn‐up not recorded). It was further distributed afterwards through three SEDS member information e‐mail dispatches reaching 1041 e‐mail addresses (although only opened by 24.6%). The potential overlap of those attending the meeting and those opening the e‐mail is unknown, why the response rate cannot be calculated. Participants were informed about the survey and its purpose. Data was collected until October 2023. As the survey did not gather any personal information or sensitive data, no ethical approval was needed.

### 2.4 Data Analysis

Data was analyzed using IBM Statistics SPSS version 29.0. Due to non‐normality of data, rating options 4 and 5 were combined into “agreeing” and options 1 and 2 were combined into “disagreeing”, while the single option 3 represented “neutral”. Descriptive data (frequencies) are presented for items in each section and in Table 1. Differences in rating patterns on items capturing respondents' attitudes (15 items) were explored using Fisher's exact test (profession; categories with < 5 individuals excluded) and Spearman correlations (*ρ*; experience, personal exercise habits). Here, findings at *p* < 0.05 were considered significant.

**TABLE 1:** Item content and responses to the survey about knowledge, clinical practices, and access to structured guidance regarding maladaptive exercise in Swedish eating disorder clinicians (*N* = 102).

Knowledge about maladaptive exercise in eating disorders					Clinical practices for maladaptive exercise in eating disorders					Access to structured guidance on maladaptive exercise in eating disorders				
Item		Options	*n*	%	Item		Options	*n*	%	Item		Options	*n*	%
12	I have good knowledge about physical activity/exercise as a symptom in eating disorders	Disagree	6	5.9	19	Patients' relationship to and attitudes toward physical activity are assessed by me or someone else at my workplace	Disagree	6	5.9	28	There is a policy/guideline for how to manage physical activity as a symptom at my workplace	Yes	43	42.2
		Neutral	19	18.6			Neutral	13	12.7			No	35	34.3
		Agree	77	75.5			Agree	82	80.4			Don't know	24	23.5
13	I think that problems around physical activity/exercise is clearly described when diagnosing eating disorders	Disagree	44	43.1	20	Patients' frequency, intensity, type, and duration of physical activity are assessed by me or someone else at my workplace	Disagree	7	6.9	29	We use BMI a basis to assess suitability of physical activity for a patient at my workplace	Yes	48	47.1
		Neutral	48	47.1			Neutral	13	12.7			No	45	44.1
		Agree	10	9.8			Agree	82	80.4			Don't know	9	8.8
14	I have received education on how to work with physical activity/exercise in eating disorder treatment	Disagree	58	56.9	21	If there is physical activity in my patients' symptomatology, I will work with physical activity as part of the therapeutical work	Disagree	4	3.9	31	There is a procedure for how to resume healthy exercise for patients who have been dissuaded from exercising during treatment at my workplace	Disagree	36	35.3
		Neutral	25	24.5			Neutral	19	18.6			Neutral	27	26.5
		Agree	19	18.6			Agree	79	77.5			Agree	38	37.3
15	I feel confident with working with physical activity/exercise in eating disorder treatment	Disagree	20	19.6	22	There is access to a physiotherapist at my workplace	Disagree	26	25.5	32	The eating disorder care would benefit from common guidelines for management and treatment of physical therapy	Disagree	3	2.9
		Neutral	35	34.3			Neutral	4	3.9			Neutral	13	12.7
		Agree	47	46.1			Agree	72	70.6			Agree	85	83.3
16	My experience is that patients with eating disorders automatically achieve a healthier relationship with exercise when they improve in their eating pathology	Disagree	61	59.8	23	Patients are offered some kind of physical activity as part of the treatment at my workplace	Disagree	53	52.0	33	There is a lack of knowledge about exercise/physical activity within the eating disorder care	Disagree	7	6.9
		Neutral	36	35.3			Neutral	22	21.6			Neutral	39	38.2
		Agree	4	3.9			Agree	27	26.5			Agree	55	53.9
17	I experience that problematic exercise tends to linger longer than other eating disorder symptoms (such as self‐induced vomiting and binge‐eating)	Disagree	13	12.7	24	Controlled and supervised physical activity can be offered during eating disorder treatment	Disagree	36	35.3					
		Neutral	28	27.5			Neutral	23	22.5					
		Agree	61	59.8			Agree	40	39.2					
18	I experience problematic exercise as an aggravating symptom for patients with eating disorders	Disagree	6	5.9	25	Physical activity during eating disorder treatment can be helpful for patients (disregard of cases where their medical status indicates physical therapy as potentially harmful)	Disagree	9	8.8					
		Neutral	9	8.8			Neutral	24	23.5					
		Agree	87	85.3			Agree	68	66.7					
					26	Physical activity during eating disorder treatment can be negative for patients (disregard of cases where their medical status indicates physical therapy as potentially harmful)	Disagree	32	31.4					
							Neutral	41	40.2					
							Agree	29	28.4					
					27	Interventions aiming to increase knowledge about physical activity and its implications in eating disorder pathology should be included in eating disorder treatment	Disagree	1	1.0					
							Neutral	3	2.9					
							Agree	97	95.1					

## 3 Results

### 3.1 Descriptive Data

**FIGURE 1:**
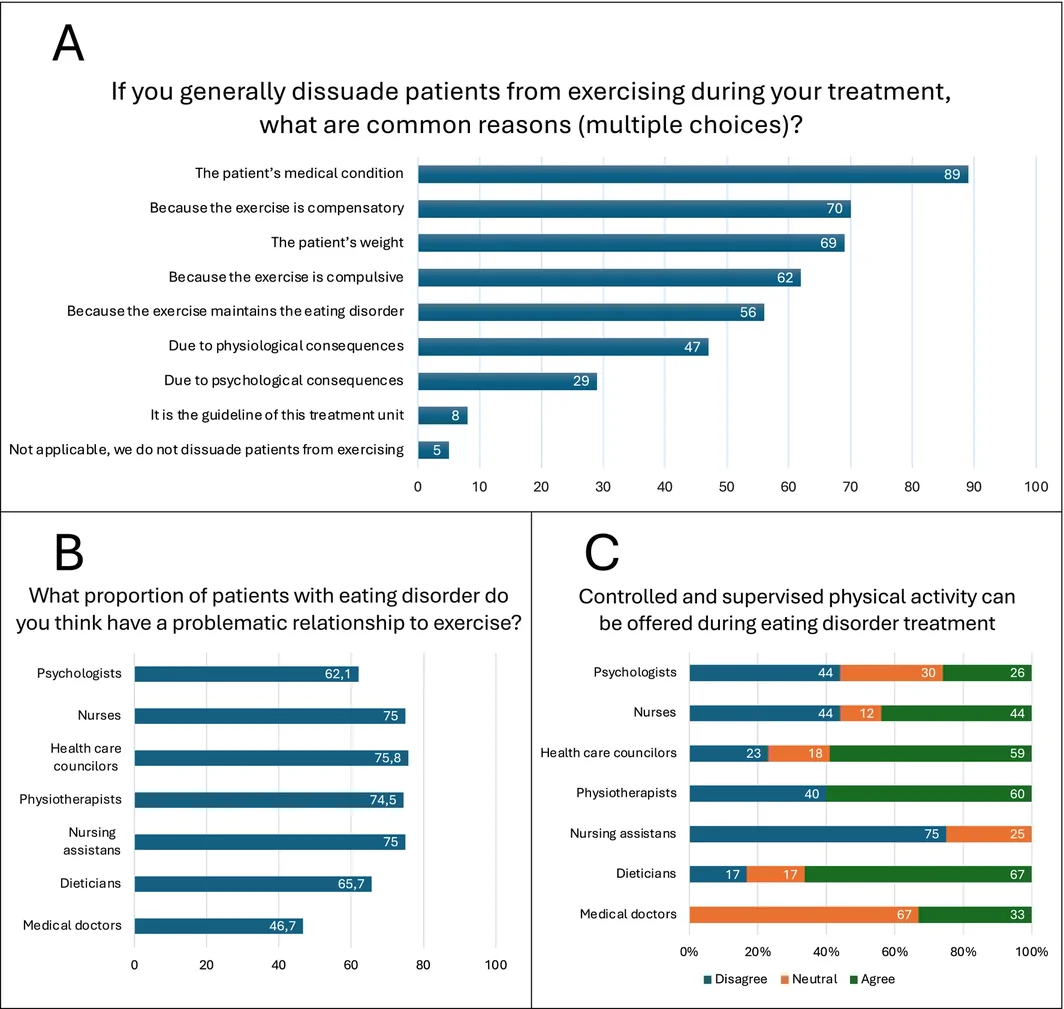
Item responses per profession capturing percentage estimation of patients with maladaptive exercise (panel A), opinion on offering exercise within treatment (panel B), and number of participants agreeing to different reasons to dissuade from exercise (panel C).

Participants were psychologists (24%), nurses (17%), health care councilors (17%), physiotherapists (10%), nursing assistants (8%), dieticians (7%), medical doctors (6%), managers (4%), and other professions (6%). Mean working experience within ED care was 9.4 years (SD = 8.9; range 0–36 years). Participants reported engaging in everyday physical activity on average 9.6 h/week (SD = 5.6; range 1–30) and in structured exercise on average 3.5 h/week (SD = 3.6; range 0–16). The participants estimated that 69% of all patients with EDs had problematic exercise (SD = 19.02; range 20%–80%). This estimation differed with profession (examined using ANOVA; *F*_6,82_ = 3.12, *p* = 0.008) with medical doctors estimating less than half, while the remaining professions estimated at least two thirds (Figure 1, panel A).

### 3.2 Knowledge About Maladaptive Exercise in Eating Disorders

A vast majority (85%) considered maladaptive exercise as an aggravating symptom; almost two thirds (60%) stated that maladaptive exercise remains longer than other symptoms and that ED improvement not necessarily entail maladaptive exercise improvement. Even though only a minority reported having received formal education about maladaptive exercise (19%) and thought it was clearly described in diagnostic systems (10%), three fourths still reported having good knowledge of maladaptive exercise in EDs. Less than half (46%) felt confident working with maladaptive exercise. More experience indicated slightly higher self‐perceived knowledge (*ρ* = 0.210, *p* = 0.01) and confidence working with maladaptive exercise (*ρ* = 0.176, *p* = 0.027). More personal structured exercise indicated slightly higher agreement with exercise being clearly described in diagnostical systems (*ρ* = 0.205, *p* = 0.04), while neither personal everyday physical activity nor profession had any associations with knowledge.

### 3.3 Clinical Practices for Maladaptive Exercise in Eating Disorders

Maladaptive exercise was largely assessed at the units (80%), and most assessed frequency, intensity, and/or duration (80%). Most (77%) reported that they address physical activity within treatment if they consider it to be a salient symptom, while respondents' personal exercise habits indicated slightly less inclination of working with maladaptive exercise (everyday physical activity: *ρ* = −0.209, *p* = 0.041; structured exercise: *ρ* = −0.216, *p* = 0.03). Most (71%) reported having access to a physiotherapist. However, controlled physical activity within treatment was rarely offered (27%) and there were differing opinions about the appropriateness of such interventions; 39% considered it appropriate, 35% considered it inappropriate, and 23% remained neutral. These opinions differed by profession (*p* = 0.008), with dieticians, physiotherapists and counselors being most positive (Figure 1, panel B). Experience showed no association with work practices.

### 3.4 Access to Clinic‐Level Practices/Informal Protocols for Maladaptive Exercise in Eating Disorders

About 42% reported having access to clinic‐level practices/informal protocols for maladaptive exercise management, while 34% had no such clinic‐level practices/informal protocols and the remaining 24% did not know if there were any. Almost half (47%) reported that certain BMI‐levels yielded exercise restriction, but that there were other factors too that could render a recommendation of exercise restriction (still medical condition/BMI as top reasons; Figure 1, panel C). Almost an equal amount perceived that their unit had (37%) versus had not (35%) clinic‐level practices/informal protocols for how to help patients resume exercise after a restriction period, while 27% were neutral. A vast majority (84%) agreed that national guidelines or protocols for interventions regarding problematic exercise would be beneficial for ED treatment, where more personal everyday physical activity indicated slightly higher agreement (*ρ* = 0.206, *p* = 0.046). Lastly, more than half (54%) considered that knowledge of maladaptive exercise was lacking within the field overall, while only 7% considered the knowledge as adequate (38% remained neutral).

## 4 Discussion

This study explored how Swedish ED clinicians understand and address maladaptive exercise, focusing on their knowledge, clinical practices, and perceptions of access to structured guidance, as well as the influence of profession, experience, and personal exercise habits. Maladaptive exercise in EDs was generally considered a widespread, long‐lasting, and aggravating symptom, though formal training in relation to it was limited, but self‐rated knowledge was high. Findings suggest that there is a readiness to identify and manage maladaptive exercise within current ED care, although the methodology, skill, and appropriateness in doing so remain unclear.

Although only a small proportion of clinicians reported formal training in maladaptive exercise, most considered themselves knowledgeable about the topic. This suggests that clinicians may rely heavily on clinical experience or informal knowledge acquisition. Still, less than half reported feeling confident working with maladaptive exercise, and even fewer found diagnostic systems to offer clear guidance. The low confidence may reflect the lack of structured training and established, evidence‐based protocols to lean against. Uncertainties around managing the symptom may contribute to many clinicians primarily recommending exercise restriction (Quesnel et al. 2018, 2025), even though its benefit is debated even in patients with severe anorexia nervosa (e.g., Ibrahim et al. 2019). Interestingly, self‐perceived knowledge and confidence increased slightly with more years of clinical experience, highlighting the likely role of experiential learning in building clinical competence in this area.

Most clinicians assessed exercise behaviors during treatment, and a majority reported addressing maladaptive exercise if deemed a salient symptom. However, interventions involving controlled physical activity were infrequently used, with mixed views on their appropriateness. These attitudes varied by profession, with physiotherapists, dieticians, and healthcare counselors being more positive toward such interventions. This suggests that professional training background likely shapes views on the therapeutic role of physical activity in ED treatment. Notably, clinicians who themselves engaged in more exercise were slightly less inclined to address maladaptive exercise, potentially reflecting an unconscious bias or normalization of high activity levels. This study highlighted a widespread assessment of exercise behaviors, with a majority assessing quantitative features (e.g., frequency, intensity). However, it is unknown whether psychological or motivational aspects—components emphasized in theoretical definitions of maladaptive exercise (Meyer et al., 2011; Sicilia et al. 2021), were just as commonly assessed. Generally, there is a need for more validated assessment tools that target both quantity and target the cognitive and emotional underpinnings of maladaptive exercise (Schaumberg et al. 2026). The lower prevalence estimation of maladaptive exercise by the medical doctors may potentially relate to higher reliance on observable or behavioral parameters (evident in some but not all patients), while for instance psychologists may be more likely to also consider the psychological underpinnings, and therefore, identify the symptoms in a higher proportion of patients. This may reflect discipline‐specific lenses through which ED symptoms are viewed and prioritized.

Access to clinic‐level practices/informal protocols for assessing or managing maladaptive exercise was inconsistent. While 42% reported having clinic‐level practices/informal protocols, an equal portion either lacked them or were unsure of their existence. Moreover, even fewer reported having clinic‐level practices/informal protocols for reintroducing exercise after restriction. These gaps point to a lack of standardized protocols and likely contribute to the uncertainty and variability in practice, as has also been pointed out by others (e.g., Davies et al. 2007; Noetel et al. 2017; Mathisen et al. 2023). Encouragingly, a strong majority of clinicians expressed a desire for national guidelines or protocols, underlining the perceived importance of structured, evidence‐based approaches and aligning with previous results (Quesnel et al. 2025). Of note, BMI and/or medical condition remained the main reason for exercise restriction (Bratland‐Sanda et al. 2009).

### 4.1 Clinical Implications

Despite being both common and serious (e.g., Liao et al. 2024; Monell et al. 2018), maladaptive exercise remains insufficiently defined and operationalized within both research and clinical guidelines (e.g., Schaumberg et al. 2026). Our findings add to this literature by demonstrating that these knowledge gaps are not only theoretical but also impact clinical practice. Clinicians in our study expressed uncertainty regarding how to define, assess, and manage maladaptive exercise, and highlighted the need for clearer guidance and more standardized approaches. Still, the vast majority reported assessing and managing maladaptive exercise. As noted, established clinical guidelines and the most common treatment protocols provide little guidance in this, and although interventions such as exercise restriction, body awareness, controlled exercise, and psychoeducation are used clinically, the empirical evidence supporting these approaches remains limited and fragmented (e.g., Bravo et al. 2024; Mathisen et al. 2023). As such, our results imply that clinicians likely rely on individual knowledge accusation, own definitions/routines, and/or clinic‐level practices/informal protocols. As such, approaches to assessment and treatment are bound to vary across settings, may lack evidence, and are potentially used arbitrarily.

Taken as a whole, there is need for clearer integration of maladaptive exercise within existing treatment frameworks. Given the profession‐related differences found in attitudes and routines, interdisciplinary training efforts may help create a more cohesive and informed clinical approach. Implementation of exercise protocols likely depends heavily on organizational culture and leadership support; in the Quesnel et al. (2025) study, clinicians in supportive settings felt more empowered to integrate physical activity into treatment, while others identified negative attitudes toward exercise as a key barrier. This suggests that successful adoption of new practices will require not just clear guidance/protocols, but also cultural and structural shifts within treatment settings.

### 4.2 Strengths and Limitations

This study offers valuable insights into how maladaptive exercise is understood and managed by a diverse group of Swedish ED clinicians. The sample included a range of professions and represented most geographic regions in Sweden, contributing to the study's relevance for national ED care. However, several limitations must be acknowledged. The data are self‐reported, which may introduce recall and social desirability biases. The survey, although informed by expert opinion and clinical experience, was not formally validated, limiting the precision and comparability of the results. Additionally, recruitment through professional networks may have introduced selection bias, potentially underrepresenting clinicians in rural or under‐resourced settings.

## 5 Conclusions

This study highlights a mismatch between clinicians' recognition of maladaptive exercise as a serious symptom that needs specific attention and the limited formal training, clinical guidance, and confidence in managing it in daily practice. There is an urgent need for clear, structured, and symptom‐specific guidance that clearly defines maladaptive exercise, offers structured assessment tools, and proposes evidence‐based interventions—including protocols for safe reintroduction of physical activity.

Future research should aim to further develop and evaluate assessment tools and intervention methods for maladaptive exercise in ED care. Longitudinal designs could explore how attitudes and routines evolve over time and in response to training and implementation of such tools and methods. Future research should also explore how different types of interventions (e.g., psychoeducation, behavioral monitoring, supervised activity) can be effectively integrated into ED care and how patients respond to such approaches.

## Author Contributions

E.M. and E.F.M. contributed equally to study preparations, data collection, study design, and manuscript writing. E.M. conducted the statistical analyses.

## Funding

The study was funded by ALF Medicine project grants (FoUI‐959,957, FoUI‐986,640), the Söderström‐Königska Foundation (SLS‐968246, SLS‐981240), and Centre for Psychiatry Research postdoc grant (2022–2023).

## Ethics Statement

The authors have nothing to report.

## Consent

The authors have nothing to report.

## Conflicts of Interest

The authors declare no conflicts of interest.

## Supporting information


**Data S1:** The full survey (35 items) was translated into English by the authors (originally in Swedish).

## Data Availability

The data that support the findings of this study are available from the corresponding author upon reasonable request.
