# Supplementary material for: Swedish Clinicians' Knowledge, Practices, and Access to Structured Guidance Regarding Maladaptive Exercise in Eating Disorders
Source: Scand J Psychol. 2026 Apr 14;67(5):1183–90. doi: 10.1111/sjop.70102 (PMC13546094; doi:10.1111/sjop.70102)
Supplement: Supplementary file 1 [file SJOP-67-1183-s001.docx]

**Supplementary materials 1 to “Swedish clinicians’ knowledge, practices, and access to structured guidance regarding maladaptive exercise in eating disorders”**

**Survey about physical activity in eating disorders**

In this survey, we would like to learn more about what you, as clinician working with patients with eating disorders, think about exercise as an eating disorder symptom. You get to answer questions about what you think of your own knowledge of problematic exercise, how you address it in treatment, and if you think that there are available guidelines concerning physical activity/exercise in eating disorders. Try to answer all questions and do so as truthfully as you can. In the end, there is a free text space where you might add other important information.

**Demography**

1. Gender
2. Age
3. What is your professional title?
4. Which (Swedish) geographic region do you belong to?
5. Do you primarily work with children/adolescents or adults?
6. Highest educational level?
   a. Upper secondary school
   b. University, BSc
   c. University, MSc
   d. University, PhD
7. For how many years have you been working with eating disorders?
8. In what type of setting do you work clinically? (multiple choices)
   a. Private practice
   b. Primary care
   c. Specialized eating disorder outpatient care
   d. Specialized eating disorder day patient care
   e. Specialized eating disorder inpatient care
   f. Emergency medical services
   g. Other: …..
9. What is your own experience of physical activity: how many hours per week do you engage in everyday physical activity (e.g., walking to/home from work, cleaning the house)? …hours per week.
10. What is your own experience of physical activity: how many hours per week do you engage in planned/structured exercise? …hours per week.
11. What proportion of patients with eating disorder do you think have a problematic relationship to exercise? Answer in percent.

**Knowledge about physical activity/exercise in eating disorders:** rated on a 5-point scale, 1=not true via 3=partly to 5=true

12. I have good knowledge about physical activity/exercise as a symptom in eating disorders
13. I think that problems around physical activity/exercise is clearly described when diagnosing eating disorders
14. I have received education on how to work with physical activity/exercise in eating disorder treatment
15. I feel confident with working with physical activity/exercise in eating disorder treatment
16. My experience is that patients with eating disorders automatically achieve a healthier relationship with exercise when they improve in their eating pathology
17. I experience that problematic exercise tend to linger longer than other eating disorder symptoms (such as self-induced vomiting and binge-eating)
18. I experience problematic exercise as an aggravating symptom for patients with eating disorders

**Working with physical activity/exercise in eating disorders:** rated on a 5-point scale, 1=not true via 3=partly to 5=true

19. Patients’ relationship to and attitudes towards physical activity are assessed by me or someone else at my workplace
20. Patients’ frequency, intensity, type, and duration of physical activity are assessed by me or someone else at my workplace
21. If there is physical activity in my patients’ symptomatology, I will work with physical activity as part of the therapeutical work
22. There is access to a physiotherapist at my workplace
23. Patients are offered some kind of physical activity as part of the treatment at my workplace
24. Controlled and supervised physical activity can be offered during eating disorder treatment
25. Physical activity during eating disorder treatment can be helpful for patients (disregard of cases where their medical status indicates physical therapy as potentially harmful)
26. Physical activity during eating disorder treatment can be negative for patients (disregard of cases where their medical status indicates physical therapy as potentially harmful)
27. Interventions aiming to increase knowledge about physical activity and its implications in eating disorder pathology should be included in eating disorder treatment

**Guidelines for physical activity/exercise in eating disorders:** rated on a 5-point scale, 1=not true via 3=partly to 5=true (exceptions in parentheses)

28 There is a policy/guideline for how to manage physical activity as a symptom at my workplace (1:yes, 2:no, 3:don’t know)
29 We use BMI a basis to assess suitability of physical activity for a patient at my workplace (e.g., no exercise/physical activity under a certain BMI; 1:yes, 2:no, 3:don’t know)
30 IF you generally dissuade patients from exercising during your treatment, what are common reasons (multiple choices):
   a. It is the guideline of this treatment unit
   b. The patient’s medical condition
   c. The patient’s weight
   d. Because the exercise is compensatory
   e. Because the exercise is compulsive
   f. Because the exercise maintains the eating disorder
   g. Due to psychological consequences
   h. Due to physiological consequences
   i. Not applicable, we do not dissuade patients from exercising
31 There is a procedure for how to resume healthy exercise for patients who have been dissuaded from exercising during treatment at my workplace
32 The eating disorder care would benefit from common guidelines for management and treatment of physical therapy
33 There is a lack of knowledge about exercise/physical activity within the eating disorder care
34 What needs do you see regarding knowledge and development related to exercise and physical activity within the eating disorder care? (free text space)
35 Free text space: Is there something more related to physical activity during eating disorders that you would like to add, that has not been covered by the questions in this survey?

Information text: would you like to share your experiences? Would you be interested of participating in a digital focus group focusing these themes to be able to elaborate your thoughts? In that case, send your name and e-mail address to [*hyperlink e-mail*]

Information text: Thank you for your participation!
